# Development and clinical deployment of a smartphone-based visual field deep learning system for glaucoma detection

**DOI:** 10.1038/s41746-020-00329-9

**Published:** 2020-09-22

**Authors:** Fei Li, Diping Song, Han Chen, Jian Xiong, Xingyi Li, Hua Zhong, Guangxian Tang, Sujie Fan, Dennis S. C. Lam, Weihua Pan, Yajuan Zheng, Ying Li, Guoxiang Qu, Junjun He, Zhe Wang, Ling Jin, Rouxi Zhou, Yunhe Song, Yi Sun, Weijing Cheng, Chunman Yang, Yazhi Fan, Yingjie Li, Hengli Zhang, Ye Yuan, Yang Xu, Yunfan Xiong, Lingfei Jin, Aiguo Lv, Lingzhi Niu, Yuhong Liu, Shaoli Li, Jiani Zhang, Linda M. Zangwill, Alejandro F. Frangi, Tin Aung, Ching-yu Cheng, Yu Qiao, Xiulan Zhang, Daniel S. W. Ting

**Affiliations:** 1 State Key Laboratory of Ophthalmology, Zhongshan Ophthalmic Center, Sun Yat-Sen University, Guangzhou, People’s Republic of China; 2 ShenZhen Key Lab of Computer Vision and Pattern Recognition, Shenzhen Institutes of Advanced Technology, The Chinese Academy of Sciences, Shenzhen, People’s Republic of China; 3 University of Chinese Academy of Sciences, Beijing, People’s Republic of China; 4 Department of Ophthalmology, The First Affiliated Hospital of Kunming Medical University, Kunming, People’s Republic of China; 5 The First Hospital of Shijiazhuang City, Shijiazhuang, People’s Republic of China; 6 Handan City Eye Hospital, Handan, People’s Republic of China; 7 C-MER (Shenzhen) Dennis Lam Eye Hospital, International Eye Research Institute of The Chinese University of Hong Kong (Shenzhen), Shenzhen, People’s Republic of China; 8 The Eye Hospital, WMU at Hangzhou, Hangzhou, People’s Republic of China; 9 Department of Ophthalmology, The Second Hospital of Jilin University, Changchun, People’s Republic of China; 10 SenseTime Group Limited, Hong Kong, People’s Republic of China; 11 Department of Ophthalmology, The Second Affiliated Hospital of Guizhou Medical University, Kaili, People’s Republic of China; 12 Department of Ophthalmology, The Second Affiliated Hospital of Xi’an Jiaotong University, Xi’an, People’s Republic of China; 13 Department of Ophthalmology, The Third Affiliated Hospital of Nanchang University, Nanchang, People’s Republic of China; 14 Hamilton Glaucoma Center, Shiley Eye Institute, Viterbi Family Department of Ophthalmology, UC San Diego, La Jolla, CA United States; 15 CISTIB Center for Computational Imaging and Simulation Technologies in Biomedicine, Schools of Computing and Medicine, University of Leeds, Leeds, UK; 16 Singapore Eye Research Institute and Singapore National Eye Centre, Singapore, Singapore

**Keywords:** Translational research, Optic nerve diseases

## Abstract

By 2040, ~100 million people will have glaucoma. To date, there are a lack of high-efficiency glaucoma diagnostic tools based on visual fields (VFs). Herein, we develop and evaluate the performance of ‘iGlaucoma’, a smartphone application-based deep learning system (DLS) in detecting glaucomatous VF changes. A total of 1,614,808 data points of 10,784 VFs (5542 patients) from seven centers in China were included in this study, divided over two phases. In Phase I, 1,581,060 data points from 10,135 VFs of 5105 patients were included to train (8424 VFs), validate (598 VFs) and test (3 independent test sets—200, 406, 507 samples) the diagnostic performance of the DLS. In Phase II, using the same DLS, iGlaucoma cloud-based application further tested on 33,748 data points from 649 VFs of 437 patients from three glaucoma clinics. With reference to three experienced expert glaucomatologists, the diagnostic performance (area under curve [AUC], sensitivity and specificity) of the DLS and six ophthalmologists were evaluated in detecting glaucoma. In Phase I, the DLS outperformed all six ophthalmologists in the three test sets (AUC of 0.834–0.877, with a sensitivity of 0.831–0.922 and a specificity of 0.676–0.709). In Phase II, iGlaucoma had 0.99 accuracy in recognizing different patterns in pattern deviation probability plots region, with corresponding AUC, sensitivity and specificity of 0.966 (0.953–0.979), 0.954 (0.930–0.977), and 0.873 (0.838–0.908), respectively. The ‘iGlaucoma’ is a clinically effective glaucoma diagnostic tool to detect glaucoma from humphrey VFs, although the target population will need to be carefully identified with glaucoma expertise input.

## Introduction

Glaucoma is the leading cause of irreversible blindness in the world, accounting for 15% of the blindness globally^1^. By 2040, it is estimated that ~100 million people will have glaucoma. Glaucoma is an optic neuropathy characterized by increased cupping of the optic disc, thinning of the neuro-retina rim with corresponding characteristic visual field (VF) defects^2^.

In clinical practice, VF can be performed in various methods, including static versus kinetic; automated versus manual and different widths of VF coverage (10 degrees, 24 degrees or 30 degrees). For detection and follow-up of glaucoma patients, the most commonly ordered VF test is 24–2 humphrey VF that covers the central 24° field^3,4^ (Supplementary Fig. 1). In brief, the VF report consists of five major maps, including numerical displays (ND), numerical total deviation plots, total deviation probability plots, numerical pattern deviation plots (NDPs) and pattern deviation probability plots (PDPs). ND shows the patient’s retinal sensitivity at specific retina region in dB (within the central 24° field). The numerical total deviation is the difference between the measured values and the age-matched controls, with the probability total deviation plot shown below. For the probability plot, it is divided into <0.5%, <1%, <2%, and <5%, with <0.5% being the most severe deviation from the normal population. For NDP and PDP, it is adjusted for general reductions in retinal sensitivity due to media opacities, uncorrected refractive error, age, and pupil size. Mean deviation (MD) is the average deviation of light sensitivity of the patients compared with age-controlled normal subjects, while pattern standard deviation (PSD) represents the irregularity of the VF by summing the absolute value of the difference between the threshold value for each test point and the average VF sensitivity at each point. Visual field index (VFI) is expressed as a percentage of visual function; with 100% being a perfect age-adjusted VF and 0% represents a perimetrically blind VF.

The interpretation of the VF reports, however, remain challenging as it requires extremely steep learning curve^5^. Many glaucoma patients were missed due to the suboptimal expertise in detecting glaucoma from VFs by general ophthalmologists or non-glaucoma specialists, especially those with early-stage glaucoma. Global parameters such as MD and PSD in VFs provide an overall picture of average glaucomatous damage; however, they rarely are aligned with the actual clinical condition nor with localized spatial information related to disease progression^6^.

To date, many groups have applied machine learning and deep learning methods in VF interpretation to detect glaucoma and predict glaucoma progression^7–12^. Most algorithms, however, are trained using single glaucoma parameter such as MD or PDP. In addition, limited study demonstrated the software development and clinical deployment of VF-based application tool for instant glaucoma diagnosis. The purpose of this study was to evaluate a multimodal VF-based deep learning algorithm, iGlaucoma, a smartphone, cloud-based glaucoma detection tool in a multi-center study.

## Results

### Patients’ demographics

A total of 1,614,808 data points of 10,784 VFs (5542 patients) from seven centers in China were included in this study. Phase I involved a total of 1,581,060 data points from 10,135 VF reports. Table 1 shows the patients’ and ocular characteristics of the glaucoma versus non-glaucoma patients. Of note, there were significant differences in age (*P* < 0.001), VFI (*P* < 0.001), MD (*P* < 0.001), and PSD (*P* < 0.001) between the two groups. Details about the demographic characteristics in different datasets are shown in the Supplementary Table 1. For Phase II, a total of 649 eyes of 437 subjects (33,748 data points) were included, and the baseline characteristics of the study subjects are summarized in Supplementary Table 2. There were significant differences in age (*P* < 0.001), VFI (*P* = < 0.001), MD (*P* < 0.001), and PSD (*P* < 0.001) between glaucoma and non-glaucoma groups. The intergrader agreement on the VF classification were substantial for both Phase I (median kappa 0.755) and Phase II (median kappa 0.824).

**Table 1:** Baseline characteristics of study participants in Phase I.

Characteristics	Non-glaucoma group	Glaucoma group	*P* value^a^
Patients (eyes)	1761 (3030)	3324 (4482)	–
Images, *n* (%)	3566 (35.2)	6569 (64.8)	–
Left/Right	1834/1732	3206/3363	–
Age, mean (SD) (years)	48.4 (17.7)	55.2 (16.4)	<0.001
VFI, median (IQR) (%)	98 (5)	91 (19)	<0.001
MD, median (IQR) (dB)	−2.78 (3.96)	−5.92 (7.58)	<0.001
PSD, median (IQR) (dB)	1.89 (1.71)	3.97 (5.99)	<0.001

*VFI* visual field index, *MD* mean deviation, *PSD* pattern standard deviation, *SD* standard deviation, *IQR* interquartile range.

^a^Comparison of the demographic and VF parameters between non-glaucoma and glaucoma groups by Wilcoxon rank sum test.

### Phase I: validation dataset

In the validation dataset of 598 VF reports for PDP (PDP-CNN), ND (ND-CNN), NDP (NDP-CNN), all CNNs achieved comparable AUCs with ND, NDP, and PDP to detect glaucoma, ranging from 0.798 to 0.862 (ND), 0.825 to 0.885 (NDP), and 0.844 to 0.900 (PDP), respectively. The corresponding sensitivities at 80% specificity were 0.704 (ND), 0.698 (NDP), and 0.740 (PDP), respectively. In the ensemble model (combining PDP, ND, and NDP), the DLS achieved the highest AUC of 0.874, with a sensitivity of 0.746 at 80% specificity. (Supplementary Table 3 and Supplementary Fig. 2).

### Phase I: test datasets

A total of 1113 VF reports from 781 subjects were included in three independent test sets. Similar to the validation dataset, the ensemble model combining PDP/ND/NDP achieved the best diagnostic performance, with a 0.873 AUC (0.822–0.924), 0.922 sensitivity (0.876–0.969), 0.676 specificity (0.567–0785) in test set 1; 0.834 AUC (0.796–0.873), 0.831 sensitivity (0.749–0.888), 0.709 specificity (0.611–0.783) in test set 2; and 0.877 AUC (0.844–0.910), 0.851 sensitivity (0.801–0.901), 0.688 specificity (0.560–0.832) in test 3. The individual results of PDP, ND and NDP were shown in Supplementary Table 4.

### Comparison of DLS vs attending ophthalmologists

**Fig. 1:**
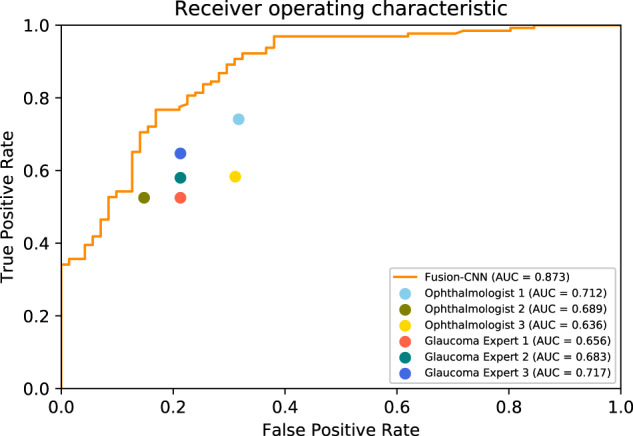
Comparison of diagnostic performance of the 2D-Fusion-CNN in VF interpretation with ophthalmologists in test set 1. The figure shows receiver operating curve of glaucoma diagnosis by the 2D-Fusion-CNN (ND + NDP + PDP) in test set 1. 2D-Fusion-CNN combining pattern deviation probability plots (PDPs), numerical pattern deviation plots (NDPs), and numeric displays (NDs) as training data outperformed all the ophthalmologists with an AUC of 0.873.

Using test dataset 1, the DLS using a combination of ND, NDP, and PDP outperformed all six ophthalmologists in detecting glaucoma (Table 2 and Fig. 1).

**Table 2:** Performance of the CNNs and ophthalmologists in test set 1.

	AUC (95%CI)	Sensitivity	Specificity	*P* value^a^
Ophthalmologists				
Attending ophthalmologist #1	0.712 (0.632–0.792)	0.741 (0.668–0.814)	0.683 (0.566–0.801)	<0.001
Attending ophthalmologist #2	0.689 (0.613–0.765)	0.525 (0.442–0.608)	0.852 (0.763–0.941)	<0.001
Attending ophthalmologist #3	0.636 (0.553–0.718)	0.583 (0.501–0.665)	0.689 (0.572–0.805)	<0.001
Glaucoma professor #1	0.656 (0.576–0.736)	0.525 (0.442–0.608)	0.787 (0.684–0.890)	<0.001
Glaucoma professor #2	0.683 (0.617–0.750)	0.580 (0.497–0.662)	0.787 (0.684–0.890)	<0.001
Glaucoma professor #3	0.717 (0.652–0.783)	0.647 (0.568–0.727)	0.787 (0.684–0.890)	<0.001
CNN				
ND + NDP + PDP	0.873 (0.822–0.924)	0.922 (0.876–0.969)	0.676 (0.567–0.785)	–
ND	0.870 (0.817–0.923)	0.915 (0.867–0.963)	0.732 (0.629–0.835)	0.81
NDP	0.857 (0.802–0.913)	0.798 (0.729–0.868)	0.817 (0.727–0.907)	0.06
PDP	0.861 (0.808–0.914)	0.868 (0.810–0.927)	0.718 (0.614–0.823)	0.06

*CNN* convolutional neural network, *ND* numeric displays, *NDP* numerical pattern deviation plots, *PDP* pattern deviation probability plots. *AUC*, area under curve.

^a^Comparison of AUC between the ND + NDP + PDP and the other groups using Z test.

### Characteristics of misinterpretations by DLS

The characteristics of the misinterpretations by the Fusion-CNN in test datasets were summarized in Supplementary Table 5. The features of false-positive results (*n* = 118) include: (1) diffuse decrease of light sensitivity caused by cataract (*n* = 87); (2) retinal diasease (*n* = 19); (3) neuro-ophthalmic diaseses (*n* = 2); (4) high myopia (*n* = 10). False-negative results (*n* = 103) were mainly due to: (1) VF of preperimetric glaucoma (*n* = 67); (2) superior/inferior peripheral scotoma (*n* = 16); (3) glaucoma with high myopia (*n* = 8); and (4) glaucoma with cataract (*n* = 12).

**Fig. 2:**
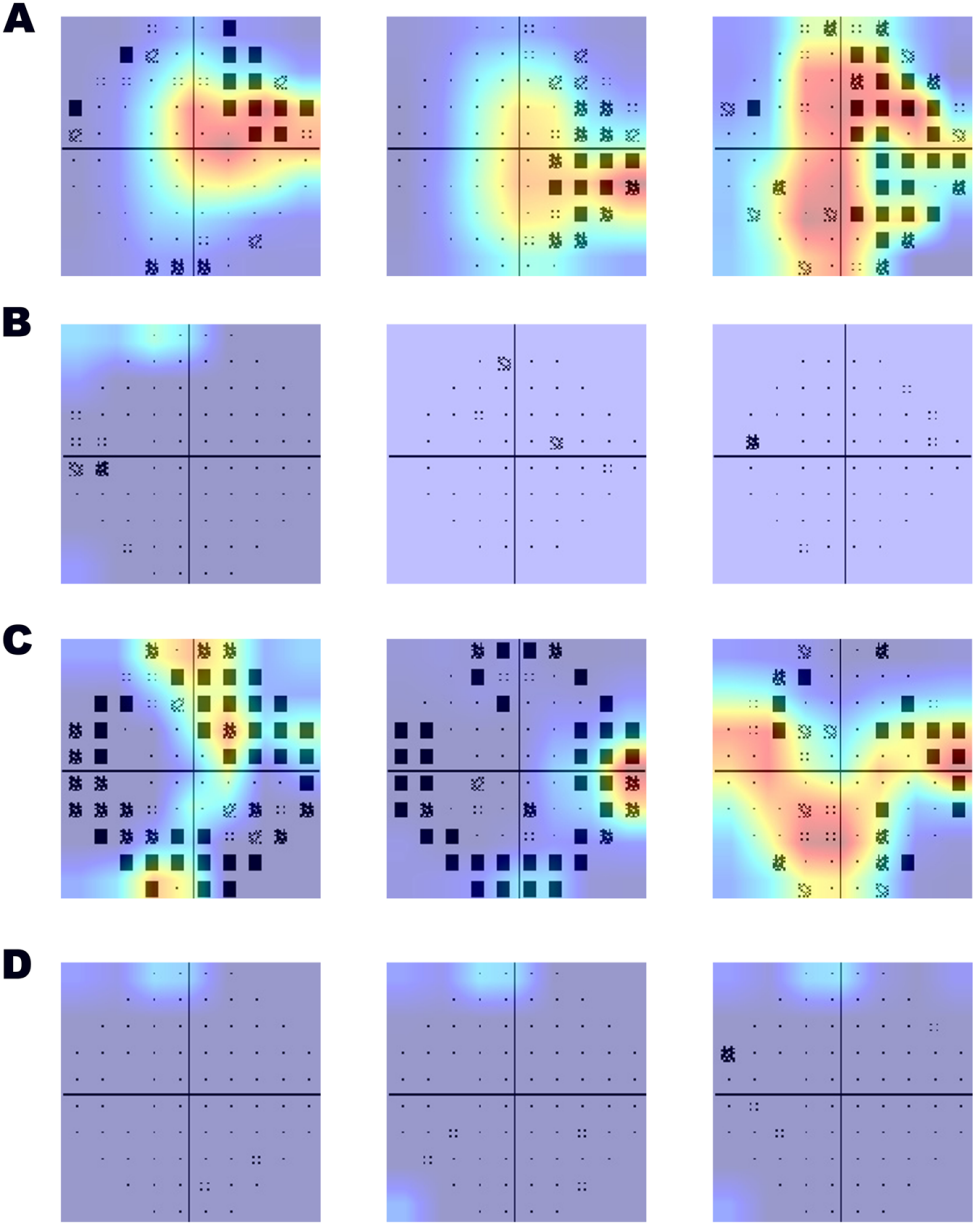
Representative heatmaps generated by the CNNs. The figure shows the heatmaps of the typical samples of eyes with and without glaucoma detected by the PDP-CNN. **a** and **b** stand for the heatmaps generated in the true-positive and true-negative cases, while **c** and **d** stand for the false-positive and false-negative cases.

Figure 2 displays the heatmaps of the typical samples of eyes with and without glaucoma detected by the DLS, and the false-positive/negative samples judged by the DLS. In the analysis of the false-positive results, the PDPs showed diffuse defects caused by cataract or retinal diseases, which is similar to the VF pattern of moderate or advanced glaucoma. The DLS focused on the defects in the VF caused by other ocular diseases and misclassified as glaucoma. For the false-negative samples, because preperimatric subjects have few defects, no heated area was identified among them.

### DLS performance stratified by age, site of eye, and mean deviation

Supplementary Table 6 and Supplementary Fig. 3 shows the results of the subgroup analysis in the validation and test sets. All DLS performances for different age groups (<60 vs 60 years or more), laterality of eye (right vs left) and severity of glaucoma (MD better than −6 dB vs −6 dB or worse) showed no statistical significance except the AUCs of the younger and older age group in test dataset 1.

### Phase II: test dataset

For clinical deployment, we developed the iGlaucoma app which can capture the printed VF reports and make diagnosis based on the captured PDP images (Supplementary Video 1**:** using the app to diagnose glaucoma VF; Supplementary Video 2**:** using the app to diagnose non-glaucoma VF). The recognition accuracy and diagnostic performance of the iGlaucoma app on printed VF reports were evaluated in this phase. First, the general recognition accuracy of different patterns on PDP map was 99.85% (Supplementary Table 7). The recognition accuracies of blank space, >5%, <5%, <2%, <1%, and <0.5% patterns were 0.999, 0.999, 0.996, 0.996, 0.995, and 1.000, respectively. Second, the software DLS application achieved an AUC of 0.966 (0.953–0.979) with a sensitivity of 95.4% and specificity of 87.3% on the PDP map, while for ophthalmologists, they were 0.850 (0.819–0.992), 85.8% (95% CI), and 84.3% (95%CI), respectively (Supplementary Table 8). The total time taken to analyze the PDP map on 549 VF reports was 9.3 min for software application, and 50.6 min for ophthalmologists.

## Discussion

In the assessment of slightly over 1.6 million data points from seven tertiary glaucoma centers in China, this study demonstrated the translation of a robust smartphone-based DLS, iGlaucoma, from bench-to-bedside in detection of glaucoma from VF over two phases. Several major findings are as below. First, the combination of PDP/ND/NDP yielded the best diagnostic performance (AUC 0.873, sensitivity 0.922, and specificity 0.676) in detecting glaucoma in Phase I, compared to the individual parameter alone. This suggests that the potential benefits of including multi-dimensional VF data to train a DLS to detect glaucoma from VF. Additionally, this level of performance, judged solely based on VFs, is more superior than all 6 general attending ophthalmologists, suggesting that this could be an useful DLS to be incorporated into the VF machine to help with decision making to diagnose the possibility of glaucoma. Second, iGlaucoma, an enhanced DLS version, is one of the world’s first smartphone-based applications that is capable of detecting glaucoma from the PDP map of humphrey VFs. It was shown to have robust and superior diagnostic performance, as compared to general ophthalmologists, to detect glaucoma on a real-world prospective dataset (Phase II), with excellent AUC (0.96), sensitivity (95.4%) and specificity (87.3%). This has high clinical utility value in helping general ophthalmologists or optometrists to diagnose glaucoma, preventing one of the major causes of irreversible blindness. Third, the time taken for iGlaucoma was five times faster than general ophthalmologists, suggesting that this could be used as one of the future glaucoma diagnostic tools in the primary eye care community, although future research is of great importance to thoroughly evaluate the cost-effectiveness of iGlaucoma. Fourth, the iGlaucoma was designed to be compatible with both iOS and Android, although the patients will still require to have Humphrey VF test performed by trained technician. Previous studies have explored the way to use virtual reality to perform perimetry at home^13,14^. In the future, if the Humphrey Field Analyzer could be transplanted into a virtual reality goggle, then we may combine the VR and deep learning techniques to create a smart VF testing and diagnostic device. This has enormous potential for mass clinical deployment for both developed and developing countries.

In this study, we also analyzed the mis-diagnosed cases by the DLS. False-negative results were mainly due to glaucoma with preperimetric changes or superior peripheral defects. Given that the gold standard diagnosis was made based on clinical, functional and structural information, it would be difficult for the DLS to detect preperimetic changes as it is trained purely-based on VF. The few cases with superior peripheral scotoma were not detected by the DLS, as these early VF changes did not follow the classic glaucoma changes (nasal steps or temporal wedges). On the other hand, false-positive results were mostly due to cataract. Although these changes were supposed to be accounted by PDP, the VF changes may still persist in patients with severe cataracts.

The diagnosis of glaucoma requires multimodal information, including clinical data (risk factors), examination findings (iridocorneal angle, intraocular pressure, central corneal thickness), structural imaging (e.g., fundus photo, optical coherene tomography (OCT) or Heidelberg Retinal Tomography) and functional imaging (VFs)^15^. Several DLS studies showed the effectiveness of fundus or OCT imaging in detecting glaucoma suspect or glaucoma^16–21^. These studies explored the diagnostic performance of using structural information in automated diagnosis of glaucoma. Interstingly, with supplement of OCT images in training data, the discriminating ability of the DLS was further enhanced, indicating the advantages of using multimodal data in developing DLS^19^.

VF-based machine learning algorithms were broadly divided into detection of glaucoma and prediction of glaucoma progression. Prior to deep learning, several studies have described using machine learning approach to detect glaucoma^22,23^. In a total of 345 eyes (156 glaucoma and 189 non-glaucoma), Goldbuam et al. reported that the Gaussian (MoG) model, among the machine learning models, yielded the highest performance (AUC: 0.923) in detecting glaucoma on 24–2 humphrey VF. With the advent of deep learning, Kucur et al. utilized numerical total deviation plots of 2267 24–2 VF samples (201 subjects) and a customized CNN to train the deep learning system^24^, showing a precision score of 0.874 that is better than other conventional machine learning models. For glaucoma progression. Yousefi et al. adopted unsupervised Gaussian mixture model to predict glaucoma progression from VF^10^. Using MD and total deviation values from 2085 VFs of 1214 subjects, the algorithm was more sensitive in detecting glaucoma progression. Additionally, Wen et al. also successfully trained a deep learning model on 32,443 VFs (4,875 patients) to forecast MD change over a 5 years’ period on VF, showing an average difference of 0.41 dB between the predicted and actual MD values^25^.

Compared to the above-mentioned algorithms, this study has several unique features. First, the study sample size is large, involving more than 1.6 million data points from 10,784 samples (5542 patients) from seven centers. Second, the ground truth was made based on multimodal data. Third, we utilized not only total deviation plots but also pattern deviation plots to enhance the performance of the algorithm. Most importantly, we have also created a software solution to clinically deploy this algorithm. For iGlaucoma, it is downloaded into the mobile application, and linked to the DLS hosted in the cloud to increase the accessibility of the AI algorithm. It is estimated that there are about 1.4 billion users of iOS and 2 billion users of Android. iGlaucoma supports most of the portable devices running these operating systems, granting it huge potential in assisting both patients and ophthalmologists in glaucoma diagnosis. Nevertheless, the access to a Humphrey VF machine, sometimes, could be an issue, especially for the under-resourced countries. Thus, it is important for the AI developers to work with the VF machine companies to lower the cost by increasing the screening uptake, potentially reducing the glaucoma-related blindness worldwide.

In Phase II, to perform an automated image analysis, the recognition of the location of the image is extremely crucial. In our study, there is a in-built “recognition algorithm” to detect the cross of PDP map, and also to automatically recognize the different pattern deviation on the PDP. Compared to general ophthalmologists, iGlaucoma has higher diagnostic performance with faster reading time. Having such assistive tool enables general ophthalmologists to be more vigilant about the early cases of glaucoma.

The performance of the DLS is better in Phase II. The main reasons for this are as follows. Firstly, all the data in Phase II were collected from the real-world eye clinics. In China, more than 80% of the glaucoma patients already had symptoms before they first went to glaucoma clinics and 75% of the patients with chronic glaucoma were identified as moderate or advanced stage of glaucoma in at least one eye at the diagnostic visit^26^. As a result, the data in Phase II contains a lot of subjects in moderate or advanced stages of glaucoma. And there would be more typical VF patterns among these patients. Secondly, in the clinics, the patients who receive 24–2 VF tests are mostly glaucoma or neuro-ophthalmic disease patients. The patients with retinal diseases don’t receive 24–2 VF tests as regular tests, and in the Phase II, the proportion of subjects with retinal diseases is lower than Phase I. This also partially contributed to the better performance of the algorithm in Phase II. Considering the above condition, the diagnostic accuracy of iGlaucoma would expect to be lower in the community where there are many more preperimetric glaucoma, glaucoma suspects or patients with VF defects from other ocular pathologies.

This study has several limitations. First, this study is limited to the Chinese population, and it is important to test this in the other ethnicities (e.g., Caucasian whites, African American, Hispanic and etc). Second, this DLS only utilizes VF, and future study will of great value to combine clinical data, examination findings and structural imagings to diagnose glaucoma. Third, we do not have patients’ long term data to develop the predictive algorithm. It is of great value to build DLS to predict the rate of glaucoma progression, or conversion of preperimetric to perimetric glaucoma, and this may be prevented by having early intervention in lowering the intraocular pressures. Fourth, iGlaucoma requires internet connection to link up with the cloud-based DLS application for glaucoma detection. Future software development is necessary to explore the possibility of deploying this DLS as an API format inside the phone. Fifth, only recognition algorithm of the PDP region was developed in Phase II. Updated algorithms able to recognize the whole VF reports but not only PDP regions are worth further investigation. Sixth, in Phase II all the patients were recruited from glaucoma clinics, where many of them had moderate to advanced glaucoma with typical VF deficits. Thus, they would have worse MD value and VF indices. Future research will be of great value to evaluate the generalizability of the DLS in the general population. Seventh, the iGlaucoma was developed based on the Humphrey Field Analyzer, which is the most widely used model in clinics and researches. Data from other models, such as Octopus by HAGG STREIT, are not supported due to different number of test locations.

In conclusion, DLS outperformed general ophthalmologists in the accuracy and timing in detecting glaucoma from VFs in the tertiary glaucoma clinics, with the best performance achieved by the combined PDP/ND/NDP algorithms. Future research is of great value to further evaluate the feasibility of using ‘iGlaucoma’, a smartphone and DLS-based application, as a screening tool in the primary eye care settings to identify early glaucoma patients who require intervention to prevent irreversible visual loss.

## Methods

### Ethical approval and study registration

The current study was approved by the Ethical Review Committee of Zhongshan Ophthalmic Center, The First Affiliated Hospital of Kunming Medical University, The First Hospital of Shijiazhuang City, Handan City Eye Hospital, C-MER (Shenzhen) Dennis Lam Eye Hospital, and The Eye Hospital, WMU at Hangzhou and The Second Hospital of Jilin University. The study was performed in accordance with the Declaration of Helsinki for research involving human subjects. Informed consent was obtained from all human participants before entering the study. The study has been registered in clinicaltrials.gov (NCT03759483, NCT03268031).

### Technical design of DLS

We exploited a convolutional neural network (CNN)—modified ResNet-18^27^, was utilized to classify the VF into glaucoma vs non-glaucoma (Supplementary Fig. 4). In brief, using optical character recognition (OCR) technique, all PDP, ND, and NDP data points were extracted from the VFs reports with intact spatial information as input data into several convolutional layers, followed by a 2-D fully-connected layer and softmax, to generate a score for glaucoma versus non-glaucoma, respectively, while applying batch normalization after the convolutional layers to reduce overfitting. All hidden layers use Rectified Linear Units (ReLUs) behind the convolution layer, which acts as nonlinear activation functions improving model. Dropout regularization is adopted to release overfitting. Lastly, the Gradient-weighted Class Activation Mapping (GradCAM) was applied to the CNN model to generate heat map on the PDP regions suggestive of glaucoma^28^, aiding the physicians to make diagnosis of glaucoma.

### Clinical datasets

**Fig. 3:**
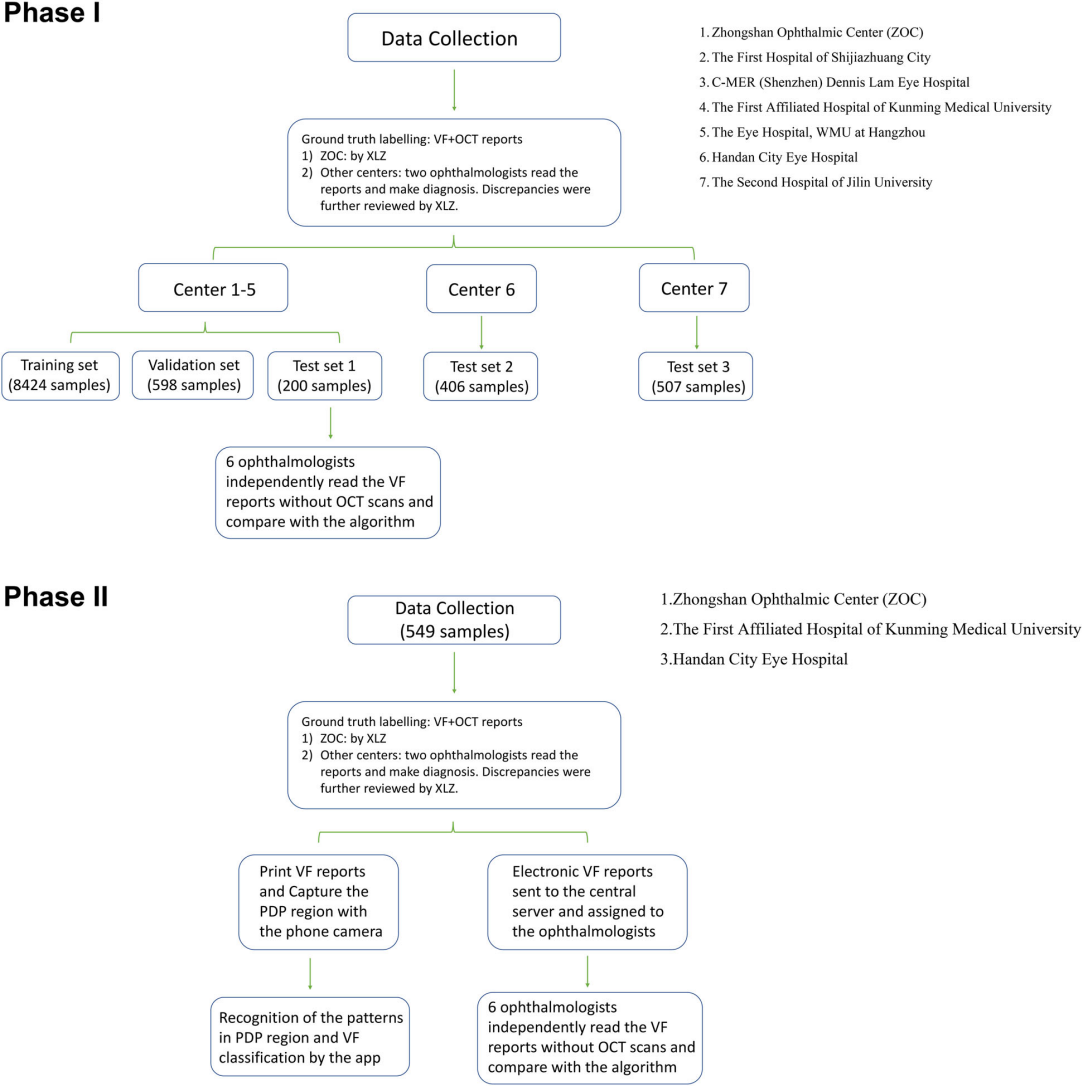
Flow chart of the current study. The study is composed of two parts. In Phase I, we developed the deep learning algorithms for classifying VFs. In Phase II, a smartphone app based on the deep learning algorithm was created and tested in the real world.

The study is divided into two phases (Fig. 3). In Phase I, to train, validate and test the diagnostic performance of the DLS in detecting glaucomatous VFs, the clinical data of both patients and normal subjects from electronic medical records or clinical research databases were collected from seven eye centers across mainland China from September 1, 2017, to March 1, 2019. Data were first divided into the training set (8424 samples from 5917 eyes of 3913 subjects) and validation set (598 samples from 586 eyes of 424 subjects), subsequently tested on three independent datasets (Test 1: 200 samples from 139 eyes of 97 subjects, Test: 2: 406 samples from 482 eyes of 372 subjects and Test 3: 507 samples from 370 eyes of 312 subjects). Phase 2 entails the development of the smartphone-based software application that incorporate the Phase 1 DLS hosted in the cloud. Using this application, a total of 649 VFs were utilized to test this smartphone integrated DLS. All the VF reports of a single subject were included in the training, valiadation or test sets to ensure these datasets were unique at patient level.

All the VFs were automated white-on-white perimetry SITA 24–2 standard/fast VFs acquired by Humphrey Field Analyzers (Carl Zeiss Meditec, Dublin, CA). Each ND, NDP, or PDP was composed of 52 data points representing different locations in the VF. The inclusion criteria in the study were: (1) All participants were ≥18 years old; (2) Study subjects had definite diagnosis of glaucoma or non-glaucoma supported by VFs, optical coherence tomography (OCT) and medical history records. Exclusion criteria of the data include: (1) VFs with fixation losses of over 2/13 or false-positive rate over 15% or false-negative rate over 25%; (2) VF reports without PD plots. If both eyes of the same subjects met the inclusion criteria, both eyes would be recruited.

VF reports were classified (i.e., ‘yes’/‘no’) according to the presence of glaucomatous optic neuropathy, including retinal nerve fiber layer (RNFL) thinning and VF defects^29^. Glaucomatous optic neuropathy was diagnosed based on the combination of VF and OCT reports. Glaucoma was diagnosed if there is a thinning RNFL correlated with VF defects in the corresponding position^30^. A glaucomatous VF defect was defined as the presence of a cluster of at least three contiguous non-edge points on the pattern deviation plot with a probability of occurring in <5% of the normal population (*p* < 0.05), with one of these points having a probability of occurring in <1% of the normal population (*p* < 0.01)^31^. Preperimetric VF reports without any deficit were excluded. All cases were evaluated in the way as mentioned above.

### Phase I: training and validation datasets

For training dataset, VF reports were acquired from 5 glaucoma clinics—Zhongshan Ophthalmic Center; The First Hospital of Shijiazhuang City; C-MER Shenzhen Dennis Lam Eye Hospital; The First Affiliated Hospital of Kunming Medical University; The Eye Hospital, WMU at Hangzhou. For validation dataset, a total of 598 VFs were randomly selected from the above eye centers. Subjects’ demographic information, clinical examination data (intraocular pressure—IOP, status of the iridocorneal angle), OCT reports and VF reports were sent to Zhongshan Ophthalmic Center and graded by two expert glaucoma specialists (FL and XYL), arbitrated by a 3rd senior glaucomatologist should there be a discordant finding (XLZ).

### Phase I: testing datasets

Test set 1 was collected from the same centers as the training and validation set with no overlap. Test set 2 and 3 were were collected from Handan City Eye Hospital and The Second Hospital of Jilin University, respectively. Based on the observations in the validation dataset (598 VFs), we tuned the model’s hyper-parameters (e.g., kernel size, stride, padding, the convolutional layer learning rate, weight decay, learning rate scheduling method of the optimizer, the number of hidden layers). Finally, the selected model is used to predict the responses in all three test datasets. The DLS diagnostic performance (on PDP, ND, NDP, and combined PDP/ND/NDP) was compared against three expert glaucomatologists (reference standards) in all three test datasets. The diagnosis of glaucoma made by these three glaucomatologists were based on clinical history, examination and investigation findings. Additionally, using test dataset 1 (200 VFs), a group of six attending ophthalmologists (three junior and three senior general ophthalmologists) were asked to diagnose glaucoma based solely on VFs, and the results were compared against the DLS.

### Phase II: real-world clinical deployment

VFs with fixation losses of over 2/13 or false-positive rate over 15% or false-negative rate over 25% or without PD plots were excluded. 13.6% of the VFs were excluded due to the above reasons. None of the eyes in Phase I was included in Phase II. From Phase I, the DLS, locked on the same operating threshold for PDP model, was further enhanced and built into a software application. An additional algorithm, “recognition algorithm”, was developed to first to detect the cross in the central of PDP map and then to determine one of the six patterns on the PDP map—blank spaces or five levels of pattern deviation probabilities—>5%, <5%, <2%, <1%, and <0.5%. To classify these six classes, a global average pooling layer with a 6-way fully-connected layer was applied, using cross entropy loss as the loss function for its characteristics.

For clinical deployment, the capturing and analysis steps are as follow: First, a phone camera (iPhone X) can be utilized to capture the printed VF reports on the PDP map on the VF printout using iGlaucoma app. The software application will automatically send the image to the remote server. Detection algorithm deployed at the server would detect the cross at the center of the PDP map using the HRNet^32^ (Supplementary Fig. 5), followed by recognition of the five deviation probabilities or the blank space using the ResNet-18. Second, information of the data points in the PDP regions would be transferred to the classification algorithm, developed in Phase I, on the remote server. Then a diagnostic result would be generated and transmitted back to the cell phone with instant diagnosis of glaucoma status.

Following the development of this software application, a total of 649 VFs of 437 patients were prospectively recruited from three glaucoma clinics between March 1, 2019 and September 1, 2019 (test dataset 4). Test dataset 4 have been graded by DLS and three ophthalmologists, and compared against the gold standard (three expert glaucomotologists). For this grading process, each ophthalmologist was asked to log into the central server to access the VF report which has been randomly separated into three parts with equal samples, followed by determination of glaucoma status. Using PDP, the DLS diagnostic performance was compared against three ophthalmologists, with reference to the three glaucoma experts’ grading.

The time taken for DLS versus three ophthalmologists’ grading was recorded. For DLS, the time was recorded between the uploading of the PDP maps to the DLS, and the generation of diagnosis; whereas for ophthalmologists, it was from the display of the VF report on the computer screen to the selection of diagnosis by the ophthalmologists.

### Statistical analyses

First, in this study, the area under curve (AUC), sensitivity and specificity with 95% confidence interval was initially calculated based on the training and validation datasets. Second, using the optimal operating threshold determined by Youden index, the DLS diagnostic performance was calculated for AUC, sensitivity and specificity on Phase I (three test datasets on PDP, ND, NDP, combination of PDP/ND/NDP) using the predetermined operating threshold (primary outcome measure). Third, the DLS performance was compared against 6 ophthalmologists on test dataset 1, with reference to three expert glaucomatologists. The Z test was used to calculate *p* values for comparison of AUCs between groups. Fourth, the misclassified VF samples were further analyzed to ascertain the respective characteristics. Fifth, the the accuracy of “recognition” algorithm was calculated for the software application in phase II. Sixth, the DLS performance was compared against three ophthalmologists, with reference to the three expert glaucomatologists on detection of glaucoma using PDP map. Seventh, the time taken to grade a VF between DLS and attending ophthalmologists were calculated. Eighth, a subgroup analysis was further performed to evaluate the DLS performance on the site of eye (left vs right), age group (<60 years vs ≥60 years), and severity of light sensitivity (MD value > −6 dB vs MD value ≤ −6 dB). All statistical analyses were performed using R software, with continuous variables being presented as means (standard deviations, SDs), or median (interquartile range). The Wilcoxon rank sum and Chi-square tests were utilized for numerical and categorical data, respectively. The level of agreement between the two graders of VF reports was evaluated using a weighted kappa statistic. All the hypotheses tested were two-sided, and we considered *p* value of less than 0.05 to be statistically significant.

### Reporting summary

Further information on experimental design is available in the Nature Research Reporting Summary linked to this paper.

## Supplementary information

Supplementary Video 1

Supplementary Video 2

Supplementary Files

Reporting Summary

## Data Availability

The datasets used in this study originated from different principal investigators in China. Upon request, the corresponding authors, X.Z. and Y.Q., can send the data request to the individual principal investigator to seek clearance from them.

## Code availability

The DLS described in this study is kept at Shenzhen Institutes of Advanced Technology, The Chinese Academy of Sciences, China. The underlying algorithm is copyrighted by Shenzhen Institutes of Advanced Technology and Zhongshan Ophthalmic Center, and will not be available to public. The iGlaucoma app can be downloaded from Apple app store or Google Play.
