# Author Correction: Development and clinical deployment of a smartphone-based visual field deep learning system for glaucoma detection

**DOI:** 10.1038/s41746-022-00585-x

**Published:** 2022-03-22

**Authors:** Fei Li, Diping Song, Han Chen, Jian Xiong, Xingyi Li, Hua Zhong, Guangxian Tang, Sujie Fan, Dennis S. C. Lam, Weihua Pan, Yajuan Zheng, Ying Li, Guoxiang Qu, Junjun He, Zhe Wang, Ling Jin, Rouxi Zhou, Yunhe Song, Yi Sun, Weijing Cheng, Chunman Yang, Yazhi Fan, Yingjie Li, Hengli Zhang, Ye Yuan, Yang Xu, Yunfan Xiong, Lingfei Jin, Aiguo Lv, Lingzhi Niu, Yuhong Liu, Shaoli Li, Jiani Zhang, Linda M. Zangwill, Alejandro F. Frangi, Tin Aung, Ching-yu Cheng, Yu Qiao, Xiulan Zhang, Daniel S. W. Ting

**Affiliations:** 1 State Key Laboratory of Ophthalmology, Zhongshan Ophthalmic Center, Sun Yat-Sen University, Guangzhou, People’s Republic of China; 2 ShenZhen Key Lab of Computer Vision and Pattern Recognition, Shenzhen Institutes of Advanced Technology, The Chinese Academy of Sciences, Shenzhen, People’s Republic of China; 3 University of Chinese Academy of Sciences, Beijing, People’s Republic of China; 4 Department of Ophthalmology, The First Affiliated Hospital of Kunming Medical University, Kunming, People’s Republic of China; 5 The First Hospital of Shijiazhuang City, Shijiazhuang, People’s Republic of China; 6 Handan City Eye Hospital, Handan, People’s Republic of China; 7 C-MER (Shenzhen) Dennis Lam Eye Hospital, International Eye Research Institute of The Chinese University of Hong Kong (Shenzhen), Shenzhen, People’s Republic of China; 8 The Eye Hospital, WMU at Hangzhou, Hangzhou, People’s Republic of China; 9 Department of Ophthalmology, The Second Hospital of Jilin University, Changchun, People’s Republic of China; 10 SenseTime Group Limited, Hong Kong, People’s Republic of China; 11 Department of Ophthalmology, The Second Affiliated Hospital of Guizhou Medical University, Kaili, People’s Republic of China; 12 Department of Ophthalmology, The Second Affiliated Hospital of Xi’an Jiaotong University, Xi’an, People’s Republic of China; 13 Department of Ophthalmology, The Third Affiliated Hospital of Nanchang University, Nanchang, People’s Republic of China; 14 Hamilton Glaucoma Center, Shiley Eye Institute, Viterbi Family Department of Ophthalmology, UC San Diego, La Jolla, CA United States; 15 CISTIB Center for Computational Imaging and Simulation Technologies in Biomedicine, Schools of Computing and Medicine, University of Leeds, Leeds, UK; 16 Singapore Eye Research Institute and Singapore National Eye Centre, Singapore, Singapore

**Keywords:** Translational research, Optic nerve diseases

Correction to: *npj Digital Medicine* 10.1038/s41746-020-00329-9, published online 22 September 2020

In this article the affiliation ‘University of Chinese Academy of Sciences, Beijing, People’s Republic of China’ for Diping Song and Han Chen was missing. The original article has been corrected.

