# Supplementary material for: Development and clinical deployment of a smartphone-based visual field deep learning system for glaucoma detection
Source: NPJ Digit Med. 2020 Sep 22;3:123. doi: 10.1038/s41746-020-00329-9 (PMC7508974; doi:10.1038/s41746-020-00329-9)
Supplement: Supplementary file 3 — Supplementary Files [file 41746_2020_329_MOESM3_ESM.pdf]

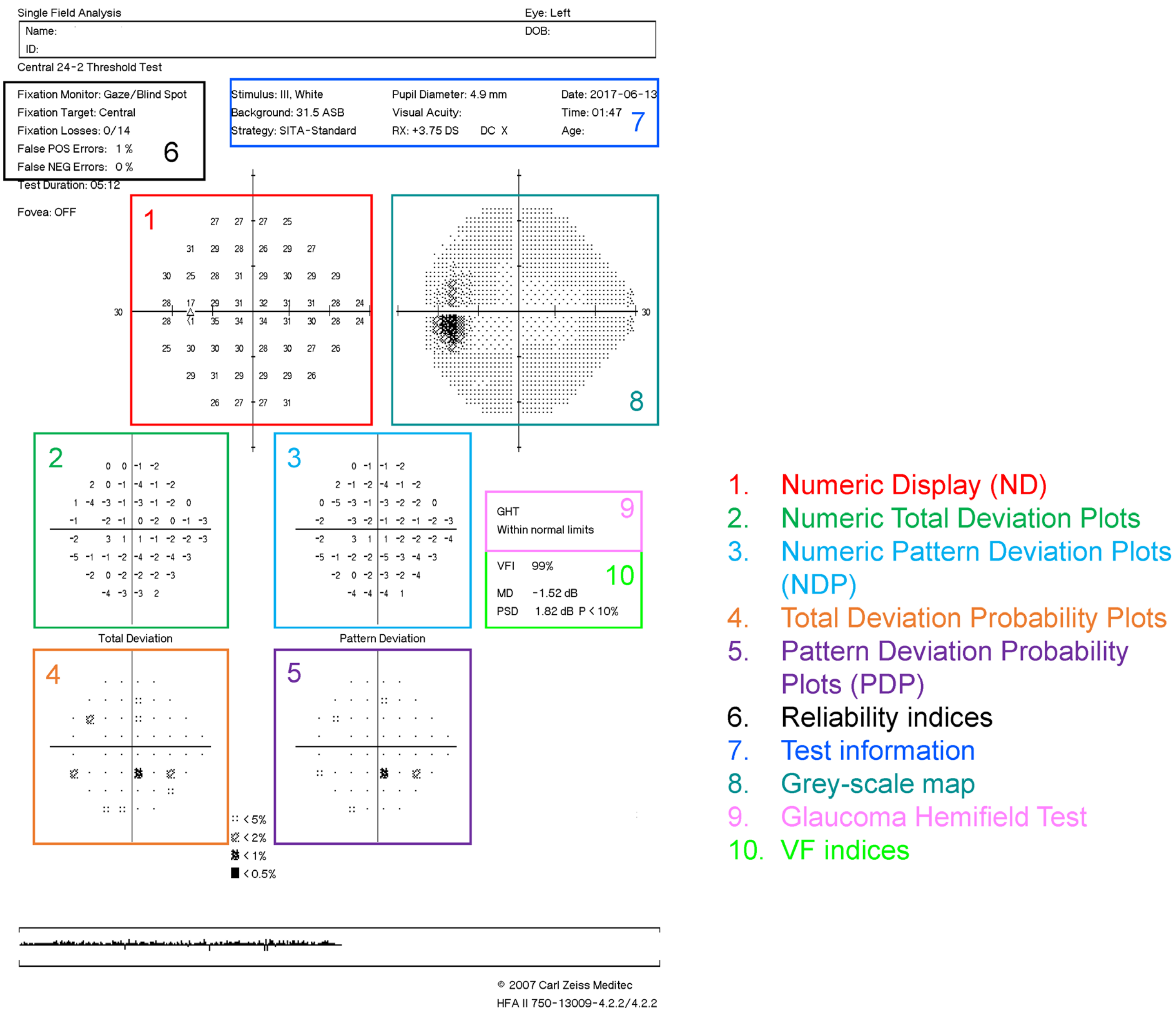

Supplementary Figure 1: Interpretation of a visual field report.

The figure displays an example of visual filed report in 24-2 mode from Humphrey Field Analyzer. It is composed of several parts: (1) Numerical display (ND) shows the patient's retinal sensitivity at specific retina region in dB; (2) Numerical total deviation plots show the difference between the measured values of light sensitivity and the age-matched controls; (4) Probability total deviation plots show the probability of the measured light sensitivity being different from the normal population; (3) & (5) For numerical pattern deviation plots (NDPs) and probability pattern deviation plots (PDPs), they display the difference and probability between the measured values of light sensitivity and the age-matched controls adjusted for general reductions in retinal sensitivity due to media opacities, uncorrected refractive error, age and pupil size; (6) Reliability indices indicate whether the visual field test is reliable; (7) This area contains the basic information of the patients, such as age, pupil size, test duration, etc; (8)

Grey scale map demonstrates regions in the visual field with decreased sensitivity in darker tones; (9) Glaucoma Hemifield Test (GHT) displays the differences between corresponding superior and inferior zones in the visual field compared with that in the population of normal controls; (10) Mean Deviation (MD) is the average deviation of light sensitivity of the patients compared with age-controlled normal subjects, while Pattern Standard Deviation (PSD) represents the irregularity of the visual field by summing the absolute value of the difference between the threshold value for each test point and the average visual field sensitivity at each point. Visual field index (VFI) is expressed as a percentage of visual function; with 100% being a perfect age-adjusted visual field and 0% represents a perimetrically blind visual field.

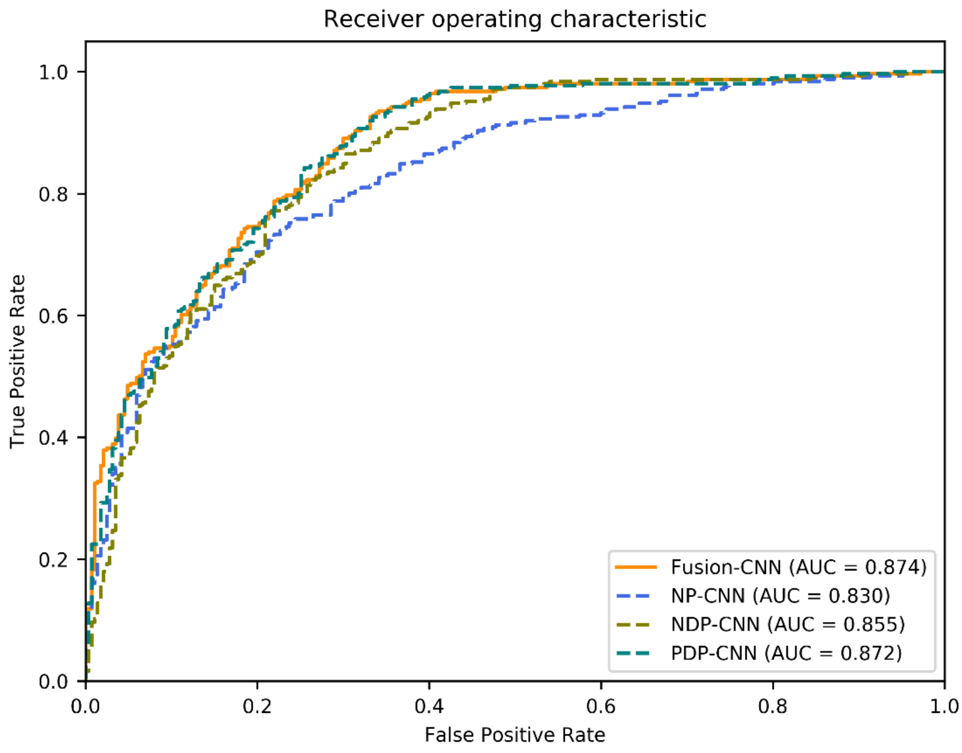

Supplementary Figure 2: Performance of the CNNs in VF interpretation in the validation set.

The figure shows receiver operating curve of glaucoma diagnosis by the CNNs in the validation set. Performance of 2D-CNNs with different input data are presented. 2D-Fusion-CNNs with the combination of pattern deviation probability plots (PDPs), numerical pattern deviation plots (NDPs) and numeric displays (NDs) as training data had the highest AUC of 0.874. CNN, convolutional neural network; ND, numeric displays; NDP, numerical pattern deviation plots; PDP, pattern deviation probability plots; AUC, area under curve.

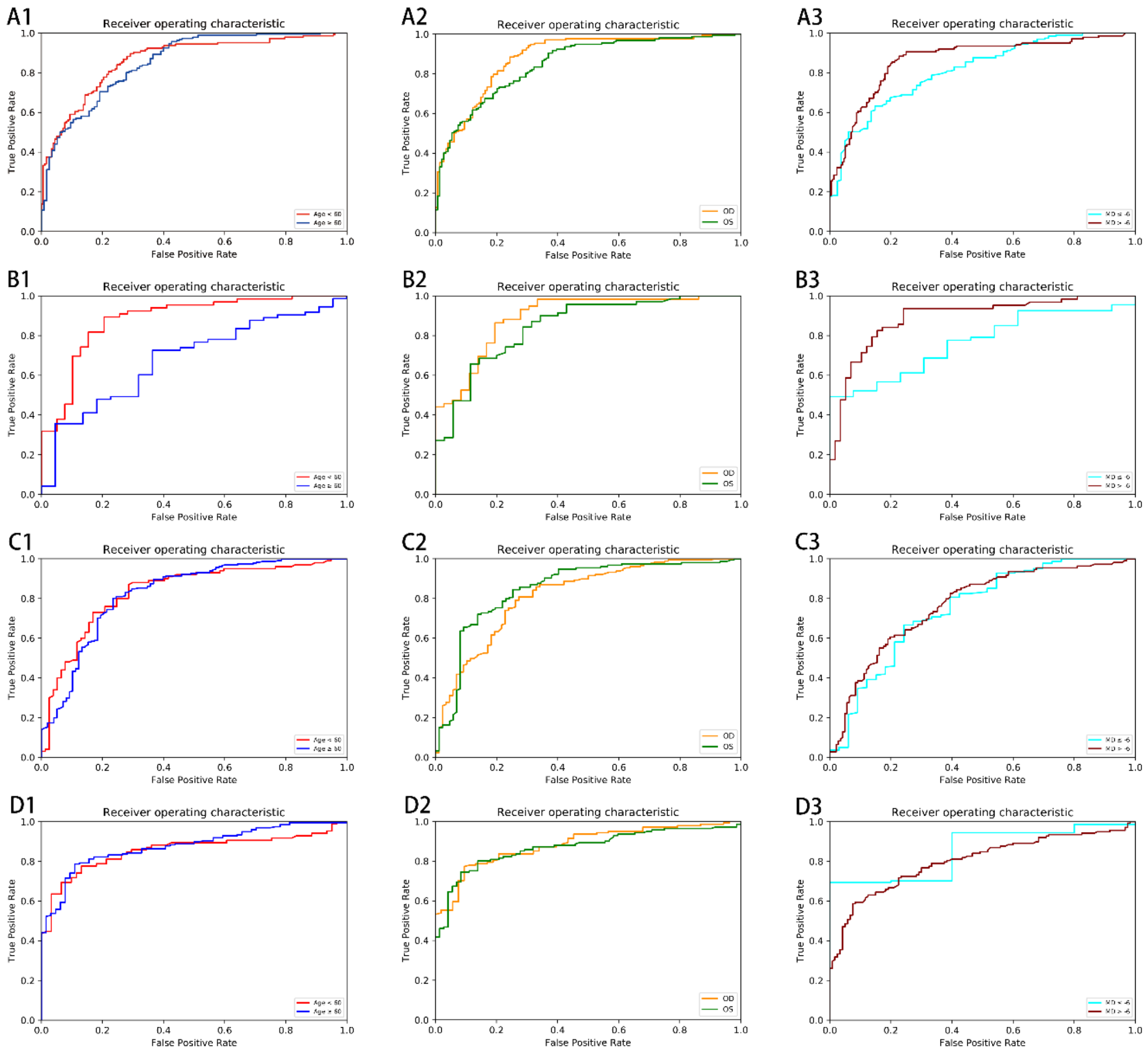

Supplementary Figure 3: Subgroup analysis of the diagnostic performance for the deep learning system (DLS) in the validation and test sets.

All DLS performances for different age groups (>60 vs 60 years or less), laterality of eye (right vs left) and severity of glaucoma (mean deviation≥-6 dB vs worse) showed no statistical significance except the AUCs of the younger and older age group in test dataset 1. A: Validation set; B: Test set 1; C: Test set 2; D: Test set 3. MD, mean deviation.

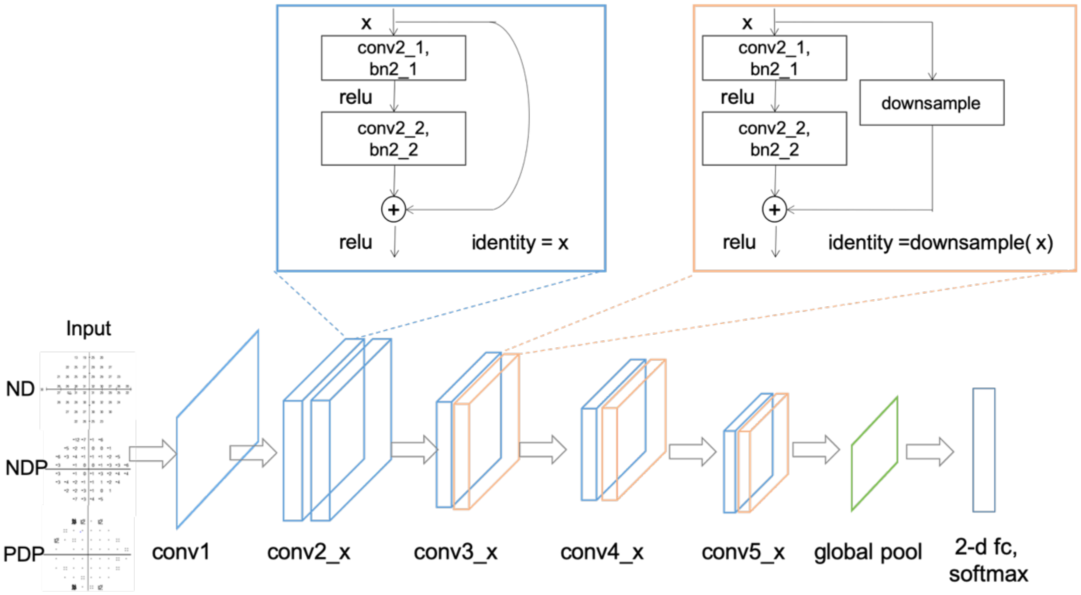

Supplementary Figure 4: Diagram of the CNN in classifying visual field reports.

The network contains 6 group learned layers: one convolutional layer, four residual learning block and 1 fully-connected layer. A global pooling layer and a softmax layer are also included. The output is fed to the softmax layer which produces a probability distribution over the 2 class labels. CNN, convolutional neural network.

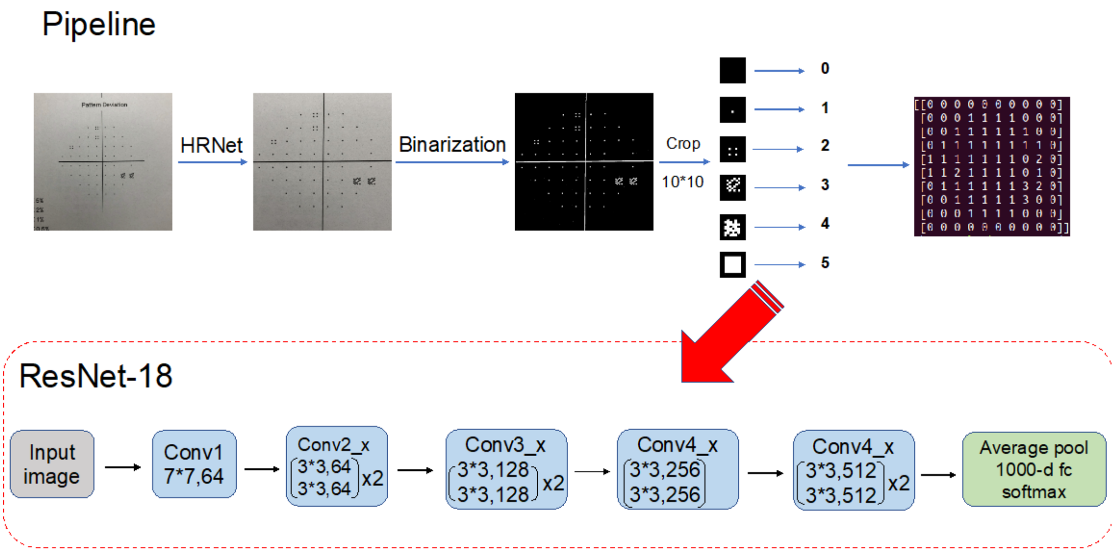

Supplementary Figure 5: Diagram of the deep learning algorithms for VF recognition

For recognition of the patterns in PDPs, we used HRNet to detect the cross in PDP region of the picture. After that, on the basis of HRNet's result, the PDP region could be detected in the pictures. Then the whole region would be cut into 100 equal-area pieces and binarized. Because there are blank spaces and five levels of pattern deviation probabilities (i.e. >5%, <5%, <2%, <1%, <0.5%) in the PDPs, the PDPs were cast into six classes. Then another deep neural network is trained based on these labeled pieces. The backbone network we choose for recognition of the patterns in PDPs is ResNet-18. In order to classify these six patterns, we add a global average pooling layer and a 6-way fully-connected layer and use cross entropy loss as our loss function for its characteristic. PDP, pattern deviation probability plots.

Supplementary Video 1: Using the app to diagnose glaucomatous VF

For clinical deployment, we developed the iGlaucoma app which can capture the printed VF reports and make diagnosis based on the captured PDP images. Users could capture the printed VF reports and upload them. After a few seconds, they will receive the diagnostic results from the server.

Supplementary Video 2: Using the app to diagnose non-glaucomatous VF

For clinical deployment, we developed the iGlaucoma app which can capture the printed VF reports and make diagnosis based on the captured PDP images. Users could capture the printed VF reports and upload them. After a few seconds, they will receive the diagnostic results from the server.

Supplementary Table 1. Baseline characteristics of study participants in different datasets of Phase I

Characteristics	Non-glaucoma Group	Glaucoma Group	P Value*
Training set			
Patients (eyes)	1,396 (2,430)	2,517 (3,487)	-
Images, n (%)	2,908 (34.5)	5,516 (65.5)	-
Left/Right	1,497/1411	2,677/2,839	-
Age(y), Mean (SD)	51.7 (17.4)	57.1 (15.7)	<0.001
VFI (%), Median (IQR)	98 (5)	92 (18)	<0.001
MD (dB), Median (IQR)	-2.80 (4.05)	-5.72 (7.38)	<0.001
PSD (dB), Median (IQR)	1.92 (1.90)	3.91 (6.15)	<0.001
Validation set			
Patients (eyes)	191 (282)	233 (304)	-
Images, n (%)	287 (48.0)	311 (52.0)	-
Left/Right	143/144	164/149	-
Age(y), Mean (SD)	50.9 (16.6)	58.9 (15.6)	<0.001
VFI (%), Median (IQR)	98 (6)	86 (25)	<0.001
MD (dB), Median (IQR)	-2.94 (4.83)	-7.48 (9.18)	<0.001
PSD (dB), Median (IQR)	1.98 (1.86)	5.31 (6.39)	<0.001
Test set 1			
Patients (eyes)	42 (59)	55 (80)	-
Images, n (%)	71 (35.5)	129 (64.5)	-
Left/Right	35/36	70/59	-
Age(y), Mean (SD)	44.6 (19.3)	59.1 (13.7)	<0.001
VFI (%), Median (IQR)	99 (2)	93 (20)	<0.001
MD (dB), Median (IQR)	-1.21 (3.33)	-6.15 (7.55)	<0.001
PSD (dB), Median (IQR)	1.55 (0.67)	3.87 (4.97)	<0.001
Test set 2			
Patients (eyes)	114 (164)	258 (318)	-
Images, n (%)	175 (34.6)	331 (65.4)	-
Left/Right	87/88	154/177	-
Age(y), Mean (SD)	57.7 (14.5)	62.7 (12.2)	<0.001
VFI (%), Median (IQR)	98 (4)	86 (18)	<0.001
MD (dB), Median (IQR)	-3.23 (3.54)	-8.44 (7.86)	<0.001
PSD (dB), Median (IQR)	1.86 (1.63)	5.15 (5.24)	<0.001
Test set 3			
Patients (eyes)	76 (110)	236 (260)	-
Images, n (%)	125 (30.7)	282 (69.1)	-
Left/Right	72/53	141/141	-
Age(y), Mean (SD)	57.8 (10.8)	62.5 (10.1)	<0.001
VFI (%), Median (IQR)	99 (1)	94 (15)	<0.001
MD (dB), Median (IQR)	-2.66 (2.28)	-6.30 (7.39)	<0.001
PSD (dB), Median (IQR)	1.74 (0.43)	3.18 (4.12)	<0.001

VFI, visual field index; MD, mean deviation; PSD, pattern standard deviation; SD, standard deviation; IQR, interquartile range

*Comparison of the demographic and VF parameters between non-glaucoma and glaucoma groups by Wilcoxon rank sum test

Supplementary Table 2. Baseline characteristics of study participants in Phase II

Characteristics	Non-glaucoma Group	Glaucoma Group	P Value*
Subjects (eyes)	201 (339)	236 (298)	-
Images, n (%)	347 (53.5)	302 (46.5)	-
Age (years), Mean (SD)	42.6 (15.0)	76.6 (17.3)	<0.001
VFI (%), Median (IQR)	99 (2)	81 (31)	<0.001
MD (dB), Median (IQR)	-0.99 (1.92)	-8.46 (9.16)	<0.001
PSD (dB), Median (IQR)	1.52 (0.51)	7.54 (7.09)	<0.001

VFI, visual field index; MD, mean deviation; PSD, pattern standard deviation; SD, standard deviation; IQR, interquartile range

*Comparison of the demographic and VF parameters between non-glaucoma and glaucoma groups by Wilcoxon rank sum test

Supplementary Table 3. Performance of the CNNs with different data input in the validation set

CNN	Input Data	AUC (95%CI)	Sensitivity 80% Specificity	85% Specificity	90% Specificity	95% Specificity	P value*
ND-CNN	ND	0.830 (0.798-0.862)	0.704	0.611	0.553	0.415	<0.001
NDP-CNN	NDP	0.855 (0.825-0.885)	0.698	0.643	0.534	0.373	0.002
PDP-CNN	PDP	0.872 (0.844-0.900)	0.746	0.669	0.582	0.469	0.63
Fusion-CNN	ND+NDP+PDP	0.874 (0.847-0.902)	0.746	0.678	0.550	0.486	-

CNN, convolutional neural network; ND, numeric displays; NDP, numerical pattern deviation plots; PDP, pattern deviation probability plots;

*Comparison of AUC between the ND+NDP+PDP and the other groups using Z test. AUC, area under curve.

Supplementary Table 4. Performance of the CNNs with different input data in the test sets

Test Set 1					
CNN	Input Data	AUC (95% CI)	Sensitivity	Specificity	P value*
ND-CNN	ND	0.870 (0.817-0.923)	0.915 (0.867-0.963)	0.732 (0.629-0.835)	0.81
NDP-CNN	NDP	0.857 (0.802-0.913)	0.798 (0.729-0.868)	0.817 (0.727-0.907)	0.06
PDP-CNN	PDP	0.861 (0.808-0.914)	0.868 (0.810-0.927)	0.718 (0.614-0.823)	0.06
Fusion-CNN	ND+NDP+PDP	0.873 (0.822-0.924)	0.922 (0.876-0.969)	0.676 (0.567-0.785)	-
Test Set 2					
CNN	Input Data	AUC (95% CI)	Sensitivity (95% CI)	Specificity (95% CI)	P value*
ND-CNN	ND	0.790 (0.748-0.831)	0.795 (0.695-0.852)	0.646 (0.697-0.846)	<0.001
NDP-CNN	NDP	0.833 (0.795-0.872)	0.822 (0.737-0.876)	0.731 (0.623-0.806)	0.86
PDP-CNN	PDP	0.825 (0.785-0.865)	0.786 (0.722-0.870)	0.714 (0.634-0.811)	0.13
Fusion-CNN	ND+NDP+PDP	0.834 (0.796-0.873)	0.831 (0.749-0.888)	0.709 (0.611-0.783)	-
Test Set 3					
CNN	Input Data	AUC (95% CI)	Sensitivity	Specificity	P value*
ND-CNN	ND	0.809 (0.766-0.853)	0.883 (0.831-0.933)	0.512 (0.352-0.640)	<0.001
NDP-CNN	NDP	0.872 (0.838-0.906)	0.777 (0.713-0.840)	0.832 (0.707-0.920)	0.36
PDP-CNN	PDP	0.867 (0.832-0.901)	0.855 (0.794-0.904)	0.672 (0.504-0.792)	0.05
Fusion-CNN	ND+NDP+PDP	0.877 (0.844-0.910)	0.851 (0.801-0.901)	0.688 (0.560-0.832)	-

CNN, convolutional neural network; ND, numeric displays; NDP, numerical pattern deviation plots; PDP, pattern deviation probability plots;

*Comparison of AUC between the ND+NDP+PDP and the other groups using Z test. AUC, area under curve.

Supplementary Table 5. The Characteristics of Misinterpretations by the CNNs in the test sets of Phase I

Types	Test dataset 1		Test dataset 2		Test dataset 3		Total
	Number	Proportion (%)	Number	Proportion (%)	Number	Proportion (%)	Number
False-Positive results	23		56		39		118
Cataract	20	87.0	40	71.4	27	69.2	87
Retinal diseases	3	23.0	14	25.0	2	5.1	19
Neuro-ophthalmic diseases	-	-	2	3.6	-	-	2
High myopia	-	-	-	-	10	25.6	10
False-Negative results	10		51		42		103
Pre-perimetric glaucoma	9	90.0	26	51.0	32	76.2	67
Superior peripheral scotoma	1	10.0	12	23.5	3	7.1	16
Glaucoma with high myopia	-	-	8	15.7	-	-	8
Glaucoma with Cataract	-	-	5	9.8	7	16.7	12

CNN, convolutional neural network

Supplementary Table 6. Comparison of AUCs of the Fusion-CNN in the validation and test sets

	Validation set			Test set 1			Test set 2			Test set 3		
	Sample size	AUC (95%CI)	P value	Sample size	AUC (95%CI)	P value	Sample size	AUC (95%CI)	P value	Sample size	AUC (95%CI)	P value
**Age**												
<60	309	0.864 (0.823,0.904)	0.74	109	0.926 (0.873,0.978)	0.004	191	0.833 (0.770,0.895)	0.87	145	0.855 (0.791,0.918)	0.59
≥60	289	0.854 (0.810,0.897)		91	0.743 (0.631,0.854)		315	0.826 (0.774,0.879)		252	0.876 (0.833,0.919)	
**Eye**												
Left	307	0.882 (0.845,0.920)	0.27	105	0.849 (0.770,0.927)	0.40	241	0.851 (0.796,0.905)	0.40	213	0.870 (0.822,0.918)	0.68
Right	291	0.851 (0.808,0.893)		95	0.893 (0.826,0.960)		265	0.818 (0.763,0.873)		194	0.884 (0.837,0.931)	
**Disease Severity**												
MD≥-6	339	0.865 (0.824,0.906)	0.13	120	0.823 (0.745,0.902)	0.33	381	0.767 (0.708,0.826)	0.93	327	0.801 (0.747,0.855)	0.43
MD< -6	259	0.813 (0.759, 0.866)		80	0.752 (0.623,0.873)		125	0.750 (0.650,0.850)		80	0.854 (0.734,0.975)	

AUC, area under curve; CNN, convolutional neural network; MD, mean deviation; CI, confidence interval；

*Comparison of AUC between groups using Z test. AUC, area under curve.

Supplementary Table 7. Recognition accuracy of the app on pictures of printed VF reports

Pattern	Total number	Recognition accuracy
Blank space	29697	0.999
>5%	22896	0.999
<5%	2449	0.996
<2%	1521	0.996
<1%	1673	0.995
<0.5%	6664	1.000

Supplementary Table 8. Performance and time cost of the deep-learning diagnostic system compared with ophthalmologists in classifying visual fields

Groups	AUC (95% CI)	Sensitivity (95% CI)	Specificity (95% CI)	Time (s)	P value*
App	0.966 (0.953-0.979)	0.954 (0.930-0.977)	0.873 (0.838-0.908)	556	<0.001
Ophthalmologists	0.850 (0.823-0.878)	0.843 (0.801-0.885)	0.858 (0.823-0.892)	3033	

*Comparison of AUC between groups using Z test. AUC, area under curve.
