# Supplementary material for: Development and clinical deployment of a smartphone-based visual field deep learning system for glaucoma detection
Source: NPJ Digit Med. 2020 Sep 22;3:123. doi: 10.1038/s41746-020-00329-9 (PMC7508974; doi:10.1038/s41746-020-00329-9)
Supplement: Supplementary file 4 — Reporting Summary [file 41746_2020_329_MOESM4_ESM.pdf]

nature research

Corresponding author(s): Daniel Ting, Xiulan Zhang, Yu Qiao, Guangxian Tang

Last updated by author(s): Jul 25, 2020

Reporting Summary

Nature Research wishes to improve the reproducibility of the work that we publish. This form provides structure for consistency and transparency in reporting. For further information on Nature Research policies, see our Editorial Policies and the Editorial Policy Checklist.

Statistics

For all statistical analyses, confirm that the following items are present in the figure legend, table legend, main text, or Methods section.

n/a	Confirmed	
☐	☒	The exact sample size (n) for each experimental group/condition, given as a discrete number and unit of measurement
☐	☒	A statement on whether measurements were taken from distinct samples or whether the same sample was measured repeatedly
☐	☒	The statistical test(s) used AND whether they are one- or two-sided<br>*Only common tests should be described solely by name; describe more complex techniques in the Methods section.*
☐	☒	A description of all covariates tested
☐	☒	A description of any assumptions or corrections, such as tests of normality and adjustment for multiple comparisons
☐	☒	A full description of the statistical parameters including central tendency (e.g. means) or other basic estimates (e.g. regression coefficient) AND variation (e.g. standard deviation) or associated estimates of uncertainty (e.g. confidence intervals)
☐	☒	For null hypothesis testing, the test statistic (e.g. F, t, r) with confidence intervals, effect sizes, degrees of freedom and P value noted<br>*Give P values as exact values whenever suitable.*
☒	☐	For Bayesian analysis, information on the choice of priors and Markov chain Monte Carlo settings
☒	☐	For hierarchical and complex designs, identification of the appropriate level for tests and full reporting of outcomes
☒	☐	Estimates of effect sizes (e.g. Cohen's d, Pearson's r), indicating how they were calculated

Our web collection on statistics for biologists contains articles on many of the points above.

Software and code

Policy information about availability of computer code

Data collection	No code was used to collect data.
Data analysis	All statistical analyses were performed using R software.

For manuscripts utilizing custom algorithms or software that are central to the research but not yet described in published literature, software must be made available to editors and reviewers. We strongly encourage code deposition in a community repository (e.g. GitHub). See the Nature Research guidelines for submitting code & software for further information.

Data

Policy information about availability of data

All manuscripts must include a data availability statement. This statement should provide the following information, where applicable:

- Accession codes, unique identifiers, or web links for publicly available datasets
- A list of figures that have associated raw data
- A description of any restrictions on data availability

The datasets used in the study are available for research purposes from the corresponding authors (Xiulan Zhang and Yu Qiao) on reasonable request. The raw data are not publicly available due to restrictions of patient privacy.

Field-specific reporting

Please select the one below that is the best fit for your research. If you are not sure, read the appropriate sections before making your selection.

☒ Life sciences ☐ Behavioural & social sciences ☐ Ecological, evolutionary & environmental sciences

For a reference copy of the document with all sections, see nature.com/documents/nr-reporting-summary-flat.pdf

Life sciences study design

All studies must disclose on these points even when the disclosure is negative.

Sample size	There was no sample size calculation. A total of 1,614,808 data points of 10,784 visual fields (5,542 patients) from 7 centers in China were included in this study.
Data exclusions	Visual fields were excluded if: (1) fixation losses of over 2/13 or false positive rate over 15% or false negative rate over 25%; (2) without pattern deviation probability plots.
Replication	The study was divided into two phases. In Phase I, a deep learning system was developed and tested in three different test sets (one internal and two external). In Phase II, the deep learning system was deployed in glaucoma clinics to validate its efficacy in glaucoma diagnosis based on visual fields in the real world.
Randomization	This is not relevant in this study as there were no different arms of the experiment.
Blinding	This is not relevant in this study as there were no different arms of the experiment.

Reporting for specific materials, systems and methods

We require information from authors about some types of materials, experimental systems and methods used in many studies. Here, indicate whether each material, system or method listed is relevant to your study. If you are not sure if a list item applies to your research, read the appropriate section before selecting a response.

Materials & experimental systems

n/a	Involved in the study	
☒	☐	Antibodies
☒	☐	Eukaryotic cell lines
☒	☐	Palaeontology and archaeology
☒	☐	Animals and other organisms
☒	☐	Human research participants
☐	☒	Clinical data
☒	☐	Dual use research of concern

Methods

n/a	Involved in the study	
☒	☐	ChIP-seq
☒	☐	Flow cytometry
☒	☐	MRI-based neuroimaging

Clinical data

Policy information about clinical studies

All manuscripts should comply with the ICMJE guidelines for publication of clinical research and a completed CONSORT checklist must be included with all submissions.

Clinical trial registration	NCT03759483, NCT03268031
Study protocol	The protocol could be reached at the website of www.clinicaltrials.gov.
Data collection	Data in Phase I were retrospectively collected from the electronic medical database in each center. Data in Phase II were collected prospectively in glaucoma clinics from three eye centers. More details can be found in the manuscript.
Outcomes	The area under curve (AUC), sensitivity and specificity of the deep learning system in glaucoma diagnosis based on visual fields.

April 2020
